# Delirium Tremens

**Published:** 1863-08

**Authors:** W. Perrie


Delirium Tremens,—Is a specific form of nervine poisoning, of which alcohol is at once the predisposing and exciting cause. The blood is poisoned by unchanged alcohol, and is rendered perfectly unfitted for the proper nutrition of the brain by long want of proper food, and by the retention of numerous matters which, in a state of health, are at once excreted, either unchanged or after undergoing some metamorphosis. Hence the proper indications for treatment are, not to trust entirely to nature, or to treat some accompanying disorder, but at once to cut off any further supply of the poisonous substance by which the blood is infected; to strive by all means to get nourishing food administered; and to take proper measures for the thorough depuration of the blood, and the elimination from the system of the many effete matters with which it is crowded. The fact of the natural subsidence of the paroxysm of delirium, quite independently of art, explains the apparent success of such different modes of treatment as have been recommended. The free exhibition of some alcoholic liquor is probably the most frequently adopted practice in this country. This treatment, though recommended by several eminent men, is not warranted by the experience of those who have had unusually large spheres of observation, Probably the profuse perspirations of delirium tremens are one of the natural means by which the depuration of the blood is effected. Hence, if the temperature becomes decidedly low, and more especially if along with this there is a decided diminution or arrest of the cutaneous exhalation, give stimulating diaphoretics, such as a mixture of camphor and ammonia.—(Dr. W. Perrie, jr.)—*Braithwaite's Retrospect.*

